# Prevention of influenza complications in patients with liver disease: a retrospective cohort study

**DOI:** 10.3389/fpubh.2023.1288126

**Published:** 2023-12-20

**Authors:** Francesco Paolo Bianchi, Francesco Losito, Nunzia Labarile, Endrit Shahini, Raffaele Cozzolongo

**Affiliations:** ^1^Epidemiology Unit, Bari Policlinico General Hospital, Bari, Italy; ^2^Gastroenterology Unit, National Institute of Gastroenterology, IRCCS S. De Bellis, Research Hospital, Castellana Grotte, Italy

**Keywords:** gastroenterology, public health, cirrhosis, infectious diseases, influenza

## Abstract

**Introduction:**

Patients with chronic liver disease are highly prone to acquiring influenza infection diseases and experiencing associated complications. National and international guidelines recommend the influenza vaccine for patients with liver disorders to reduce the risk of influenza complications. Our study aims to evaluate the risk of flu complications faced by patients with liver disease and assess influenza vaccination coverage.

**Methods:**

The archive of hospital discharge forms was used to define the list of Apulian patients with liver disease, considering data from 2017 through 2022. The vaccination status of these patients was assessed via data collected from the Regional Immunization Database. We focused on influenza vaccine shots administered during the 2020/21, 2021/22, and 2022/23 flu seasons.

**Results:**

A declining trend across the flu seasons was observed, with a VC of 49.5% in the 2020/21 flu season, 48.1% in the 2021/22 season, and 45.0% in the 2022/23 season. Subjects with multiple comorbidities have higher vaccination rates. Additionally, the multivariate models demonstrate that vaccination compliance increases with age and is strongly associated with having received a previous influenza vaccine shot.

**Conclusion:**

The VC rates reported in our study are unsatisfactory and did not reach the minimum achievable goal (75%) the Italian Ministry of Health set. A multifactorial approach is required to raise the immunization rates and therefore protect the patients from the influenza-associated risk of collateral liver damage; the role of gastroenterologists and hepatologists is crucial, as their responsibilities should extend beyond patient care to the prevention of complications after infectious diseases.

## Introduction

Individuals with liver disease are more susceptible to infections due to various pathophysiological mechanisms. These mechanisms include intestinal dysbiosis, increased bacterial translocation, portosystemic shunting, and cirrhosis-associated immune dysfunction. Cirrhosis-associated immune dysfunction is a complex condition characterized by dysregulation of both the adaptive and innate immune systems and continuous immune system activation. Additionally, factors such as malnutrition and the use of immunosuppressive medications in liver transplant recipients contribute to a heightened risk of infections in patients with liver disease (1).

Consequently, patients with chronic liver disease are highly prone to acquiring vaccine-preventable diseases (VPDs) and experiencing associated complications. In particular, influenza infection is associated with an unfavorable prognosis in patients with liver disease. A global pooled analysis in 2009 revealed that individuals with liver disease infected with the 2009 influenza A virus (H1N1) had a 5-fold higher likelihood of hospitalization due to influenza-related complications and a 17-fold higher mortality risk than healthy individuals (2). A multicenter study conducted between 2013 and 2014 reported a two-fold increased risk of hospitalization, specifically for influenza virus in patients with liver disease (3). Moreover, influenza infection can lead to hepatic decompensation in patients with cirrhosis (4).

The influenza vaccine can help mitigate the risk of such complications. A prospective multicenter study in 2018, conducted on liver transplant recipients, showed that individuals who received the influenza vaccine were less likely to develop pneumonia or require admission to the intensive care unit if they contracted laboratory-confirmed influenza A (5). Due to the high risk of severe health complications from influenza infection, individuals with liver disease are often the target of influenza vaccination policies. The US Centers for Disease Control and Prevention (CDC) and the European Centre for Disease Prevention and Control (ECDC) both recommend the influenza vaccine for patients with liver disorders to reduce the risk of influenza complications (6, 7). In Italy, at the beginning of each flu season, the Italian Ministry of Health determines the categories considered at higher risk of influenza complications based on recommendations from international public health institutions. Influenza vaccination is actively and freely offered to these high-risk categories, including individuals with underlying conditions that increase the risk of influenza-related complications, such as liver diseases. The vaccination coverage (VC) objectives in Italy were set as follows: a minimum achievable goal of 75% and an optimal goal of 95%. These goals indicate the desired percentage of the population within the target categories that should receive the influenza vaccine (8). However, despite these recommendations, low vaccine coverage is reported among Italian patients with liver disease. Stroffolini et al. (9) focused on the 2020/21 flu season and reported an overall vaccine coverage of 39.6% among cirrhotic patients. The coverage was higher in individuals older than 64 years (51.9%) and lower in those younger than 65 years (26.9%). In a 2023 study evaluating VC in patients with liver disorders aged 6 months to 64 years living in Puglia, southern Italy, during the 2020/21 flu season, a coverage rate of 24% was reported (10).

Our study aims to assess the vaccination coverage (VC) of the flu vaccine in patients with liver disease living in Puglia, Southern Italy (4,000,000 inhabitants), and track the trend from the 2020/21 flu season to the 2022/23 flu season. The estimation of VC enables us to determine the proportion of individuals at risk of flu complications and assess any measures necessary to mitigate this risk.

## Methods

This is a retrospective observational study. The study population was identified using the archive of hospital discharge forms (SDO) from Castellana Grotte “De Bellis” research and treatment hospital specializing in gastroenterological diseases. This database contains all information on hospital and inpatient procedures (10). We included all records related to cirrhosis (ICD9 codes 571.5, 571.2), liver disease (ICD9 codes: 571.49, 573.3, 570, 571.1, 070.1, 070, 571.49, 571.40, 573.3, 573.8, 572.0), hemochromatosis (ICD9 code: 275.0), Pompe disease (ICD9 code: 271.0), alpha-1 antitrypsin deficiency (ICD9 code: 273.4), steatosis (ICD9 code: 571.8), Wilson’s disease (ICD9 code: 275.1), Budd-Chiari syndrome (ICD9 code: 453.0), and hepato-cholangio- carcinoma (ICD9 codes: 155.0, 155.1), extending our search to all procedures performed from 2017 to 2022. We only considered subjects living in Apulia.

The influenza vaccination status of patients was assessed using the Regional Immunization Database (GIAVA) (10). We focused on influenza vaccine shots administered during the 2020/21, 2021/22, and 2022/23 flu seasons. The Edotto platform (Exprivia, Apulia, Italy) was used to identify deceased individuals in Apulia from 2017 to 2023, to exclude subjects who died before the start of each flu season. Information on chronic diseases was obtained from the SDO archive to characterize our sample further. Ten comorbidities were defined: chronic lung diseases, cardiopathies, diabetes mellitus and other metabolic diseases, chronic renal failure/adrenal insufficiency, hematopathies and hemoglobinopathies, tumors, HIV and immunodepression, chronic inflammatory diseases and bowel malabsorption syndromes, multiple pathologies, and dementia.

These data sources were extracted and matched using the patients’ unique identification numbers (PINs). The final dataset was created as an Excel spreadsheet that included sex, age at the start of influenza vaccination campaigns, number of comorbidities, and influenza vaccine administration before the 2020/21, 2021/22, and 2022/23 flu seasons. Anonymized data analysis was performed using STATA MP18 software. The study was carried out in accordance with the Declaration of Helsinki.

Continuous variables are presented as mean ± standard deviation and range, categorical variables are presented as proportions with 95% confidence intervals (95%CI) where applicable. A multivariate logistic regression model was built to analyze the factors associated with receiving a dose of flu vaccine prior to at least one of the analyzed flu seasons, with sex (male vs. female), age (in years), and the number of comorbidities as determinants. Another model was built to evaluate the association between influenza vaccine uptake before the 2022/23 influenza season and having received at least one flu shot before the previous seasons, adjusted for sex, age, and the number of comorbidities. Adjusted odds ratios (aORs) and 95% CIs were calculated. The Hosmer-Lemeshow chi-squared test was used to assess the goodness-of-fit of the multivariate logistic regression models.

A two-sided *p*-value < 0.05 was considered statistically significant for all tests.

## Results

**Figure 1:**
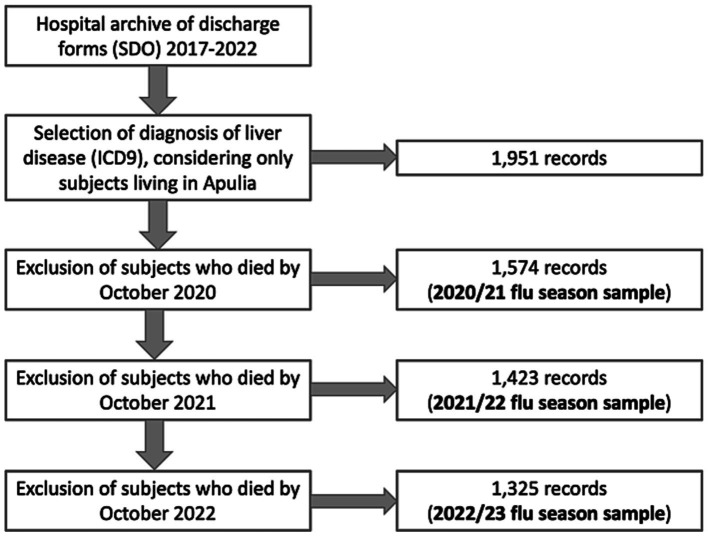
Sample size computation.

The process of sample selection in the study is depicted in Figure 1. The initial sample of eligible subjects before the 2020/21 flu season comprised 1,574 individuals with liver disease. In the 2021/22 season, the sample size decreased to 1,423 subjects; in the 2022/23 season, it further decreased to 1,325 subjects.

The characteristics of the study samples are detailed in Table 1.

**Table 1:** Characteristics of the sample.

Characteristic	2020/21 flu season	2021/22 flu season	2022/23 flu season
Sample size	1,574	1,423	1,325
Male; *n* (%)	933 (59.3)	837 (58.8)	766 (57.8)
Age at the start of the vaccination campaign (years); mean ± SD (range)	65.4 ± 14.1 (14–95)	65.5 ± 14.1 (15–96)	66.0 ± 14.1 (16–97)
**Age class; *n* (%)**			
0–17	4 (0.3)	1 (0.1)	1 (0.1)
18–49	201 (12.8)	181 (12.0)	168 (12.7)
50–64	465 (29.5)	429 (30.2)	383 (28.9)
65+	904 (57.4)	812 (57.1)	773 (58.3)
**Number of comorbidities; *n* (%)**			
0	523 (33.2)	452 (31.8)	422 (31.9)
1	150 (9.5)	144 (10.1)	140 (10.5)
2	358 (22.7)	337 (23.7)	313 (23.6)
3+	543 (34.6)	490 (34.4)	450 (34.0)

2020/21, 2021/22, 2022/23 flu seasons.

**Figure 2:**
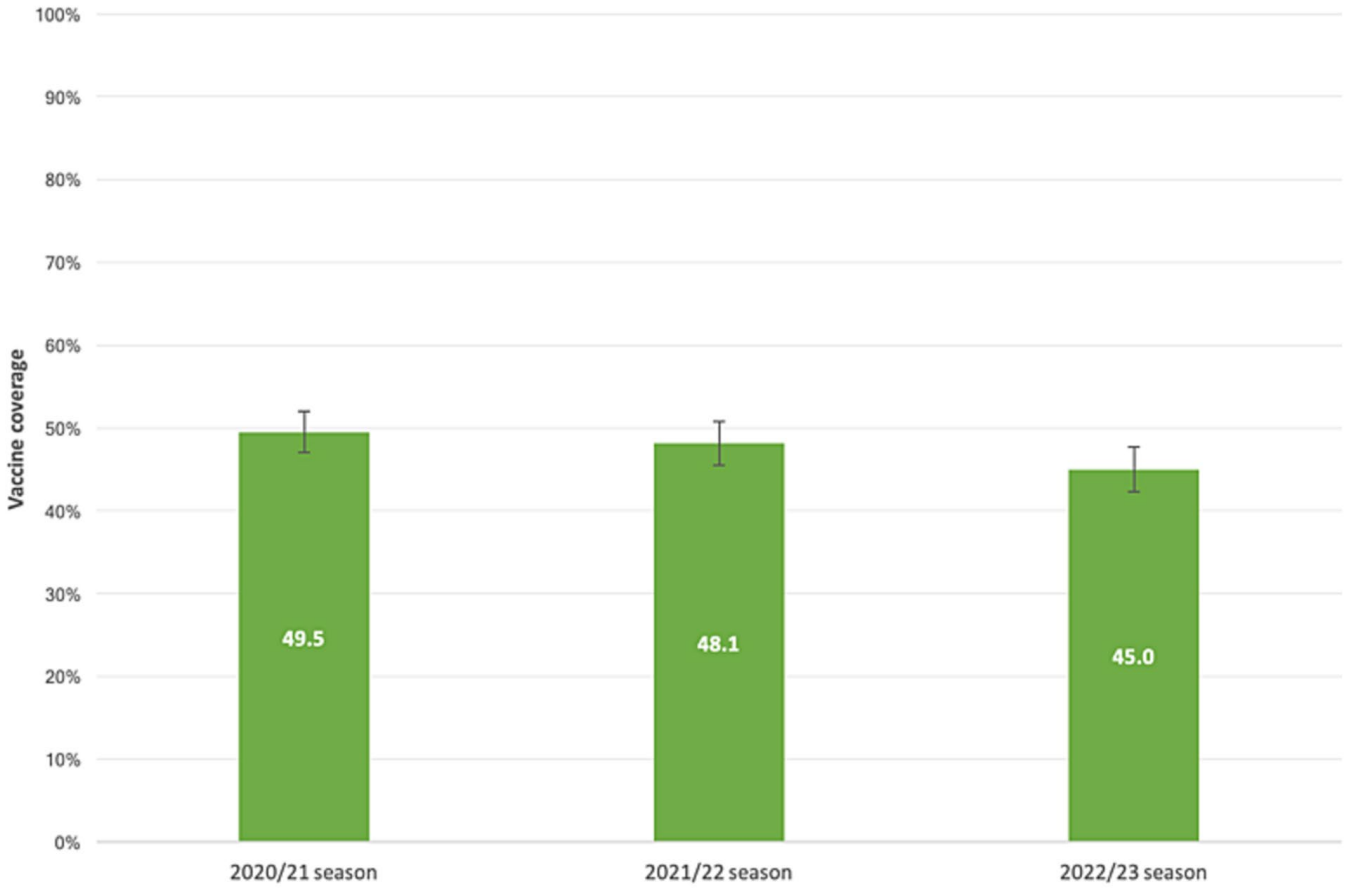
Influenza vaccine coverage in patients with liver disease. 2020/21, 2021/22, 2022/23 flu seasons.

Our samples’ VC demonstrated a declining trend across the flu seasons, as illustrated in Figure 2. Among the subjects, 936 individuals (59.5%; 95%CI = 57.0–61.9%) received at least one dose of the vaccine over the three flu seasons. Overall, 441 patients (28.0%; 95% CI = 25.8–30.3%) received the flu shot prior to all three seasons analyzed, 242 (15.4%; 95% CI = 13.6–17.3%) received the vaccine before two seasons, 253 (16.1%; 95% CI = 14.3–18.0%) received it prior to one season, and 638 (40.5%; 95% CI = 38.1–43.0%) never received a flu shot.

The values of VC, stratified per age class, and number of comorbidities are reported in Table 2.

**Table 2:** Influenza vaccine coverage in patients with liver disease, per several characteristics.

Characteristic	2020/21 flu season	2021/22 flu season	2022/23 flu season
**Sex; *n* (VC%; 95%CI)**			
Female	315 (49.1; 45.2–53.1)	273 (46.6; 42.5–50.7)	241 (43.1; 39.0–47.3)
Male	464 (49.7; 46.5–53.0)	412 (49.2; 45.8–52.7)	355 (46.3; 42.8–49.90)
**Age class; *n* (VC%; 95%CI)**			
0–17	0 (0.0; 0.0–60.2)	0 (0.0; 0.0–97.5)	0 (0.0; 0.0–97.5)
18–49	35 (17.4; 12.4–23.4)	34 (18.8; 13.4–25.2)	22 (13.1; 8.4–19.2)
50–64	164 (35.5; 30.9–39.8)	137 (31.9; 27.5–36.6)	124 (32.4; 27.7–37.3)
65+	580 (64.2; 60.9–67.3)	514 (63.3; 59.9–66.6)	450 (58.2; 54.6–61.7)
**Number of comorbidities; *n* (VC%; 95%CI)**			
0	178 (34.0; 30.0–38.3)	162 (35.8; 31.4–40.5)	141 (33.4; 28.9–38.1)
1	68 (45.3; 37.2–53.7)	53 (36.8; 28.9–45-2)	48 (34.3; 26.5–42.8)
2	190 (53.1; 47.8–58.3)	164 (48.7; 43.2–54.1)	150 (47.9; 42.3–53.6)
3+	343 (63.2; 59.0–67.2)	306 (62.5; 58.0–66.8)	257 (57.1; 52.4–61.7)

2020/21, 2021/22, 2022/23 flu seasons.

The logistic multivariate regression analysis indicated that older age (aOR = 1.05; 95%CI = 1.04–1.06) and having three or more comorbidities (aOR = 2.24; 95%CI = 1.51–3.23) were associated with receiving a flu shot prior to at least one flu season. The other factors examined did not demonstrate a significant association with the outcome (*p* > 0.05; Table 3).

**Table 3:** Analysis of the determinants of a flu shot prior to at least one flu season in a logistic multivariate regression model.

Determinant	aOR	95%CI	*p*-value
Sex (male vs. female)	1.04	0.83–1.30	0.739
Age (years)	1.05	1.04–1.06	<0.0001
**Comorbidities**			
0 vs. 1	0.77	0.52–1.13	0.176
2 vs. 1	1.42	0.94–2.13	0.092
3+ vs. 1	2.24	1.51–3.23	<0.0001

Goodness-of-fit chi2 = 6.4; *p*-value = 0.607.

Having received at least one flu shot prior to the 2020/21 and 2021/22 flu seasons appeared to be a strong predictor of receiving a flu shot prior to the 2022/23 influenza season (aOR = 26.31; 95% CI = 18.18–38.08; Table 4).

**Table 4:** Analysis of determinants of receiving a dose of influenza vaccine during the 2022/23 influenza season in a logistic multivariate regression model.

Determinant	aOR	95%CI	*p*-value
Sex (male vs. female)	1.22	0.91–1.63	0.185
Age (years)	1.03	1.02–1.04	<0.0001
**Comorbidities**			
0 vs. 1	0.82	0.48–1.41	0.480
2 vs. 1	1.19	0.79–1.80	0.399
3+ vs. 1	1.15	0.80–1.66	0.458
Flu shot in at least one previous season (YES/NO)	26.31	18.18–38.07	<0.0001

Goodness-of-fit chi2 = 10.8; *p*-value = 0.212.

## Conclusion

Our study revealed a vaccination coverage (VC) rate in our subgroup population ranging from 45 to 50% across the flu seasons. The highest VC value was observed during the 2020/21 season, which can be attributed to the co-circulation of SARS-CoV-2 and the vigorous promotion of influenza vaccination as an essential public health measure. This was particularly important considering the strain on healthcare systems during the COVID-19 pandemic, and promoting influenza vaccination helped mitigate the crisis’s health effects and social consequences. However, these VC values did not reach the minimum achievable goal (75%) the Italian Ministry of Health set. Previous studies on cirrhotic patients in Italy have reported low VC rates, with an overall VC of 39.6% in 2019, lower in people younger than 65 years of age (26.9%) compared to those 65 years and older (51.9%; *p*-value < 0.001) (9). Another study published in 2023 reported a VC of 24.0% among Apulian patients with liver disease aged 6 months to 64 years during the 2020/21 flu season (10). A study in Austria conducted in 2021 on 516 patients found that 43.9% of them expressed willingness to be vaccinated before the 2020/2021 flu season, compared to VC rates of 25.4 and 27.3% in the 2019/2020 and 2016/2018 flu seasons, respectively. These values align with our findings, as higher VC rates were observed among patients 65 years and older (57–63%), while lower rates were recorded in younger subjects (26–30%) (11).

The analyzed data show that subjects with multiple comorbidities have higher vaccination rates, as confirmed by the multivariate regression model. This finding is consistent with other studies in the literature (10). Additionally, the multivariate models demonstrate that vaccination compliance increases with age and is strongly associated with having received a previous influenza vaccine shot. A 2021 study on liver transplant patients further supported the association between flu shot uptake and previous influenza vaccination history, reporting an aOR of 20 (95% CI = 8–52) (11). This finding is consistent with several other studies in the literature that also demonstrate this association among various high-risk subgroups (12, 13).

In summary, our study highlights the need for more significant efforts to achieve higher VC in this population subgroup. It should be noted that liver disease progression is associated with immune dysregulation, and complications from influenza can result in significant morbidity and mortality, making vaccination a priority for these individuals regardless of age. Although not directly targeting the liver, influenza infection can cause collateral liver damage, such as mild and self-limiting hepatitis, and trigger hepatic decompensation in liver disease (4, 14). Furthermore, a 2019 study identified influenza infection as a trigger for the development of acute-on-chronic liver failure (ACLF), occurring in almost every fifth patient with liver cirrhosis hospitalized due to influenza infection. The study reported that influenza infection led to organ failures, secondary infections, and death, emphasizing the importance of influenza vaccination in this population (15).

Several factors contribute to the low VC values, including a lack of prevention culture among patients, challenges in implementing effective and clear vaccine communication and promotion campaigns by government and public health institutions, fear of adverse events following immunization, and unfounded concerns about worsening clinical conditions after vaccination, all of which contribute to vaccine hesitancy. It is crucial to emphasize the role of physicians, particularly gastroenterologists, and hepatologists, as patients often rely on them as trusted sources of information (9–11). They are responsible for recommending vaccination to protect patients from serious flu complications. Therefore, physicians must receive adequate training and stay updated on seasonal flu vaccine recommendations. Solid evidence supports influenza vaccination’s safety, immunogenicity, and effectiveness in patients with liver disease. A 2019 meta-analysis demonstrated that patients with liver disease mount an efficient antibody response to influenza vaccination, reducing hospital admission risk (from 205/1,000 to 149/1,000, risk difference −0.06, 95%CI = −0.07 to 0.04). Vaccinated patients were 27% less likely to be admitted to the hospital than unvaccinated patients (risk ratio 0.73, 95% CI 0.66–0.80) (16).

The strength of our study lies in its investigation of a topic that has received limited attention in the literature, employing a large sample size and examining data from three flu seasons. The study design allowed us to assess the cause-effect relationship between vaccine uptake and various determinants. However, it is essential to acknowledge that the data sources were initially established for administrative purposes rather than epidemiological research, which introduces the potential for bias. Furthermore, the data sources used in our study do not allow further analysis of vaccine effectiveness or surveillance of adverse events following immunization in this population. Future studies should consider evaluating the role of other vaccines, such as the anti-pneumococcal vaccine, and assessing vaccine coverage values over multiple influenza seasons and with larger sample sizes.

The VC rates reported in our study are unsatisfactory, and similar findings have been observed among other infectious high-risk subgroups in Apulia. According to the PASSI survey, the VC among Apulian individuals aged 18–64 years with at least one chronic disease was 38.6% during the 2019/2020 influenza season (17). A study conducted in 2023 on 1,576 splenectomized patients in Apulia showed an influenza VC of 49% for at least one flu shot after splenectomy (18). These results emphasize the need for enhanced immunization strategies to improve vaccination coverage in this subset of patients. A multifactorial approach is required, involving coordination among various healthcare workers involved in the care of patients with liver disease, including general practitioners, public health physicians, and specialists. In this regard, the role of gastroenterologists and hepatologists is crucial, as their responsibilities should extend beyond patient care to the prevention of complications after infectious diseases. The exacerbation and decompensation of a chronic patient lead to increased hospitalizations and workload for specialists, a worsening clinical picture, and additional costs for the community. Hospital facilities should also be involved in immunization strategies by actively offering vaccination prophylaxis to chronic patients, as this has been a successful strategy described in several studies in the literature (19, 20). In conclusion, preventing infectious risks in patients with liver disease must be integrated into their treatment pathways. Reducing the risk of complications leads to better patient management, improved quality of life, and better responses to treatments and care. By prioritizing influenza vaccination in this vulnerable population, we can significantly reduce the incidence of influenza-related hospitalizations, mortality, and liver-related complications.

## Data availability statement

The data analyzed in this study is subject to the following licenses/restrictions: Data are not available due to restrictions e.g. privacy or ethical. Requests to access these datasets should be directed to dr.francesco.bianchi@gmail.com.

## Ethics statement

Ethical approval was not required for the study involving humans in accordance with the local legislation and institutional requirements. Written informed consent to participate in this study was not required from the participants or the participants’ legal guardians/next of kin in accordance with the national legislation and the institutional requirements.

## Author contributions

FB: Conceptualization, Supervision, Writing – original draft. FL: Investigation, Writing – original draft. NL: Data curation, Formal analysis, Writing – original draft. ES: Validation, Writing – original draft. RC: Methodology, Writing – original draft.
